# Effects of physical activity participation on cognitive impairment in older adults population with disabilities

**DOI:** 10.3389/fpubh.2024.1293023

**Published:** 2024-01-24

**Authors:** Seung-Taek Lim, Hyo-Bum Kwak, Ju-Hee Kang, Eunwook Chang, Kyung-Lim Joa, Hee-Jung Park, Dong-Ho Park

**Affiliations:** ^1^College of General Education, Kookmin University, Seoul, Republic of Korea; ^2^Waseda Institute for Sport Sciences, Waseda University, Saitama, Japan; ^3^Institute of Sports and Arts Convergence (ISAC), Inha University, Incheon, Republic of Korea; ^4^Department of Kinesiology, Inha University, Incheon, Republic of Korea; ^5^Department of Biomedical Science, Program in Biomedical Science and Engineering, Inha University, Incheon, Republic of Korea; ^6^Department of Pharmacology and Research Center for Controlling Intercellular Communication, College of Medicine, Inha University, Incheon, Republic of Korea; ^7^Department of Physical and Rehabilitation Medicine, College of Medicine, Inha University, Incheon, Republic of Korea

**Keywords:** disabilities, older adults, cognitive function, physical activity, health

## Abstract

**Background:**

Existing research on the association between cognitive function and physical activity in the older adults population with disabilities is limited. Additionally, there is a need to explore avenues for enhancing the longevity and quality of life among these individuals.

**Objective:**

This study aimed to investigate the independent and joint associations between cognitive function and levels of physical activity in the older adults population with disabilities.

**Methods:**

A total of 315 older adults adults (men = 182, women = 133), identified with disabilities based on medical evaluation, were recruited from the first survey of the Korean Longitudinal Study of Aging (KLoSA). Participants underwent assessments for cognitive function, physical activity (PA), activities of daily living (ADLs), instrumental activities of daily living (IADLs), and grip strength.

**Results:**

ADLs (*p* < 0.001) and IADLs (*p* < 0.001) scores were significantly higher in the male normal cognitive group compared to both the male and female cognitive impairment groups. In an unadjusted model, disabled older adults individuals who did not meet the recommended PA guidelines showed an increased odds ratio for cognitive dysfunction (OR = 2.29, 95% CI = 1.32–3.97). Those participating in PA at least 1 day per week also demonstrated an elevated odds ratio (OR = 1.22, 95% CI = 1.08–1.38) for cognitive dysfunction compared to those who engaged in regular PA. A negative correlation was observed between K-MMSE scores and grip strength (*r* = 0.448, *p* < 0.001).

**Conclusion:**

This study provides robust evidence that disabled older adults individuals who do not meet the recommended guidelines for PA or who do not participate in PA at least once a week have an increased likelihood of cognitive impairment compared to those who are regularly active.

## 1 Introduction

Disability in the older adults is associated with adverse health outcomes, elevated health costs, and diminished quality of life (1). Advances in life expectancy among the older adults have been accompanied by a decrease in the duration of life lived with disability. This phenomenon, known as compressed disability, has been observed across all groups, irrespective of sex, age, and educational level, and is statistically significant (2).

A similar trend of increased prevalence has been noted for both disability and cognitive impairment (3). Cognitive function substantially influences the daily activities of older individuals, exacerbating their risk of frailty, disability, and mortality (4). Furthermore, the impact of cognitive impairment on disability is more pronounced than that of depression in this demographic (5). Additionally, low quality of life ratings for activities of daily living (ADLs) and instrumental activities of daily living (IADLs), which are used as assessments related to independent living in the older adults, had a significant impact on the prevalence of ADL and/or IADL older adults with disabilities (6). Compared to the older adults without disabilities, the older adults with disabilities were found to be at an increased risk for an increasing number of diseases. For ADL disability, the odds ratio (OR) (95% confidence interval [CI]) values for four or more diseases were 4.10–6.59, and for IADL disability, the OR (95% CI) values were 2.55–4.85 (7). Therefore, interventions are imperative for mitigating cognitive decline among the older adults with disabilities.

Various strategies exist for mitigating cognitive decline in the older adults, and increasing physical activity and physical fitness has been identified as particularly beneficial for older adults, whether they have disabilities or not. A 10-min increase in moderate to vigorous physical activity (MVPA) has been shown to correlate with lower rates of disability, indicating that higher MVPA levels can help reduce the incidence of disability in older populations (8). Individuals engaging in physical activity at a rate of 18.1 metabolic equivalent (MET)-hour/week or higher were found to have a 52% reduced risk of being classified as having a disability related to dementia or requiring care when compared to inactive individuals (9). Furthermore, older adults individuals who achieved the recommended MVPA level of 150 min per week exhibited superior cognitive function relative to those who failed to meet this standard; the latter group was 1.63 times more likely to experience cognitive decline (10). Furthermore, in both genders of the older adults, higher age, lower cognitive function, lower gait speed, and lower grip strength were predictors of difficulty living with disability (11). Over a 10-year follow-up of 1,096 participants with a mean age of 69.4 ± 5.8 years, the group with the lowest grip strength had a significantly greater decline in cognitive function compared to the group with the highest grip strength (estimate = 0.06, *p* = 0.039), indicating that low grip strength can predict 10 years of cognitive decline (12).

Nevertheless, the literature lacks comprehensive studies exploring the correlation between cognitive function and physical activity levels in older adults individuals with disabilities. For this demographic, physical activity has a statistically significant impact on annual medical expenditures, with inactive individuals incurring greater costs compared to their active counterparts (13). Therefore, it is crucial to provide the older adults with disabilities options for maintaining long-term health and wellness. This study aims to investigate the distinct and shared connections between cognitive function and physical activity levels in older adults individuals with disabilities.

## 2 Methods

### 2.1 Participation

**Figure 1:**
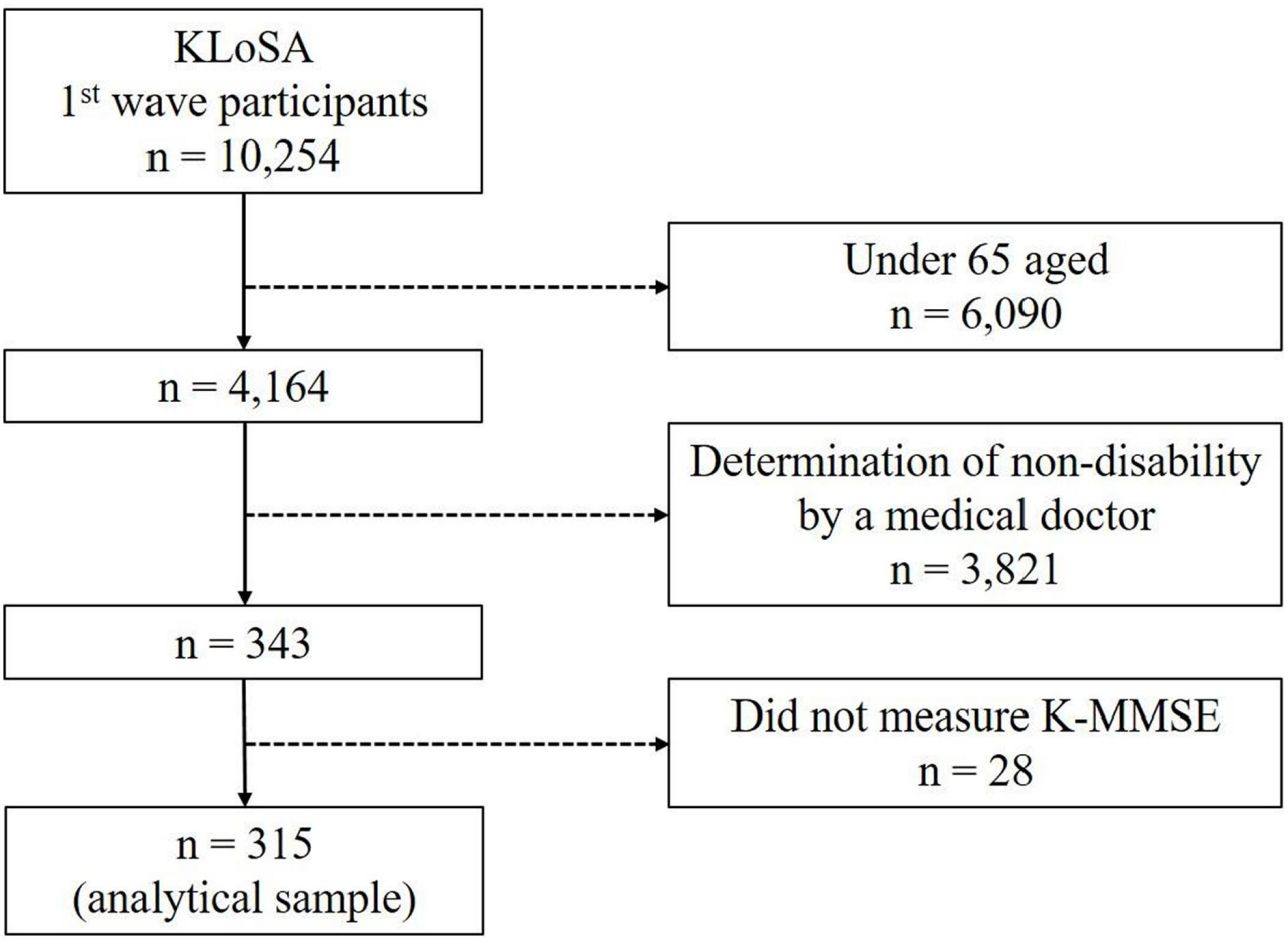
A flowchart detailing study sample selection.

The data for the present analysis were obtained from the first survey of the Korean Longitudinal Study of Aging (KLoSA). A multistage, stratified probability sampling technique was employed by the Ministry of Labor of Korea to randomly select household units based on geographical regions, including both urban and rural settings. The final survey cohort comprised 10,254 individuals, which constitutes 0.07% of the Korean population aged 45 years and above. This sample exhibited representative age and gender distributions and was geographically dispersed across 16 major metropolitan cities and provinces. For the purposes of the current study, the analysis was restricted to 315 participants (men = 182, women = 133) aged 65 years and older, who met specific criteria for medical disability (Table 1) and had available scores for the Korean version of the Mini-Mental State Examination (K-MMSE). A total of 9,939 individuals were excluded from the analysis (Figure 1).

**Table 1:** Medical determination of disability.

Variables	Male		Female	
	Normality	Cognitive impairment	Normality	Cognitive impairment
Crippled disorder	66 (59.46)	45 (59.21)	33 (71.74)	60 (59.41)
Brain lesion	9 (8.11)	10 (13.16)	3 (6.52)	9 (8.91)
Visual disturbance	8 (7.21)	9 (11.84)	5 (10.87)	11 (10.89)
Hearing defect	14 (12.61)	5 (6.57)	3 (6.52)	13 (12.87)
Dysphasia	1 (0.90)	1 (1.32)	1 (2.17)	2 (1.98)
Renal disorder	4 (3.60)	1 (1.32)	1 (2.17)	3 (2.97)
Cardiopathy	8 (7.21)	2 (2.63)	0 (0.00)	1 (0.99)
Mental retardation	1 (0.90)	3 (3.95)	0 (0.00)	2 (1.98)
Total	111 (100)	76 (100)	46 (100)	101 (100)

Case, n (%).

Ethical approval for the KLoSA was granted by its research ethics committee. The survey data are publicly accessible and can be downloaded from the designated employment survey website with personal identifiers removed. All participants in the study provided written informed consent for participation and for the use of their data for research objectives. The study was conducted in accordance with the ethical standards set forth in the Declaration of Helsinki.

Table 2 outlines the physical characteristics of the study participants.

**Table 2:** Characteristics of the study participants.

Variables	Total (*n* = 315)			
	Male (*n* = 182)		Female (*n* = 133)	
	Normality (*n* = 111)	Cognitive impairment (*n* = 71)	Normality (*n* = 40)	Cognitive impairment (*n* = 93)
Body composition, (mean ± SD)				
Age (years)	70.23 ± 4.46	73.52 ± 6.20	69.18 ± 3.52	72.98 ± 5.61
Height (cm)	167.39 ± 6.41	164.69 ± 6.36	155.45 ± 5.00	153.91 ± 5.65
Weight (kg)	64.78 ± 10.64	59.75 ± 8.98	59.45 ± 7.12	55.51 ± 9.99
BMI (kg/m^2^)	23.10 ± 3.48	22.10 ± 2.76	24.63 ± 2.91	23.38 ± 3.59
Cognitive scales, (mean ± SD)				
K-MMSE (score)	27.12 ± 1.94	17.86 ± 5.74	26.43 ± 1.65	15.80 ± 5.86
Health-related factors, (n (%))				
Smoking, n (%)	72 (64.86)	36 (50.70)	1 (2.50)	5 (5.38)
Alcohol, n (%)	79 (71.17)	18 (25.35)	7 (17.50)	13 (13.98)
Hypertension, n (%)	44 (39.64)	28 (39.44)	21 (52.50)	46 (50.55)
Diabetes, n (%)	16 (14.41)	19 (26.76)	10 (25.00)	18 (19.35)
Cancer, n (%)	6 (5.41)	2 (2.82)	0 (0.00)	0 (0.00)
Lung, n (%)	12 (10.81)	4 (5.63)	0 (0.00)	7 (7.53)
Liver, n (%)	2 (1.80)	0 (0.00)	1 (2.50)	0 (0.00)
Cardiac, n (%)	12 (10.81)	8 (11.27)	2 (5.00)	11 (11.83)
Cerebrovascular, n (%)	13 (11.71)	18 (25.35)	5 (12.50)	11 (11.83)

BMI, body mass index; K-MMSE, Korean-Mini Mental State Examination.

### 2.2 Cognitive function

For the assessment of cognitive function, we utilized panel data along with the Korean version of the Mini-Mental State Examination (K-MMSE) scores. The K-MMSE is a 30-item instrument designed to evaluate various cognitive domains including orientation, registration, attention, calculation, memory, language, and visuospatial ability. Based on a cutoff score of 24 points in the K-MMSE, study participants were categorized into two distinct groups: those with cognitive impairment (scores <24 points) and those without (scores ≥24 points).

### 2.3 Physical activity

Physical activity (PA) was assessed using a self-reported questionnaire that captured information in three specific domains: total PA time per week (in minutes), duration of each PA session (in minutes), and frequency of PA sessions per week (in days). Participants who answered “yes” to engaging in PA were further prompted to provide details on the frequency and duration of their weekly PA. The volume of PA was subsequently estimated by multiplying the weekly frequency by the duration of each session in minutes. Data on PA were collected by KLoSA and were classified as either sufficient (more than 150 min per week) or insufficient (less than 150 min per week), as per global recommendations (14, 15).

### 2.4 ADLs and IADLs

The Korean Activities of Daily Living (ADL) scale consists of seven items that assess basic daily life functions such as dressing, washing, bathing, eating, mobility, grooming, and toileting. The Instrumental Activities of Daily Living (IADL) scale evaluates the ability to perform more complex tasks such as household chores, meal preparation, laundry, short-distance travel, use of transportation, shopping, financial management, telephone use, and medication management. Each item on both scales was scored as either 1 for partial or total dependence or 0 for independence. The ADL scale ranges from 0 to 7 points and the IADL scale ranges from 0 to 10 points, with higher scores indicating lower levels of independence among the older adults population.

### 2.5 Grip strength

Grip strength was assessed using a hand grip dynamometer (NO6103, TANITA, Japan). Measurements were conducted with the participant’s elbows flexed at 90°, for both the right and left hands. The average grip strength was computed based on measurements from both hands. In cases where a subject was unable to complete the grip test with one hand, the value obtained from the other hand was used for analysis (16).

### 2.6 Statistical analysis

Results are reported as mean ± standard deviation and case percentage (%). Data analysis was conducted using SPSS version 25.0 (SPSS Inc., Chicago, IL, United States). Binary logistic regression analyses were carried out to investigate the independent and joint effects of PA time and PA frequency on cognitive function. Odds ratios (ORs) and 95% confidence intervals (CIs) were calculated for these relationships. The reference group for the joint association analyses comprised individuals who met the recommended PA guidelines, namely 150 min per week and activity on at least one day. Covariates were adjusted in the analyses and included sex (male and female), body mass index (< 25 and ≥ 25), smoking status (smoker and non-smoker), and alcohol consumption (drinkers and non-drinkers). Additional analyses were conducted to identify significant differences between sexes (male and female) in terms of group categorization (normal cognitive function and cognitive impairment) and other variables (PA frequency per week, PA time per day, grip strength, ADLs, and IADLs) using a one-way ANOVA. Correlations between K-MMSE scores and grip strength were assessed using Pearson’s correlation coefficients. The statistical significance level was set at *p* < 0.05.

## 3 Results

### 3.1 Physical activity time and frequency, grip strength, ADLs, and IADLs

Table 3 presents the PA time and frequency, grip strength, ADLs, and IADLs.

**Table 3:** Physical activity time, grip strength, ADL, and IADL scores for each group.

Variables	Male (*n* = 182)			Female (*n* = 133)		
	Normality (*n* = 111)	Cognitive impairment (*n* = 71)	*p*-value	Normality (*n* = 40)	Cognitive impairment (*n* = 93)	*p*-value
The number of PA (times/week)	5.31 ± 1.97	5.10 ± 2.23	0.695	5.89 ± 1.83	6.27 ± 1.03	0.523
PA time (min)	64.51 ± 38.83	41.76 ± 26.27	0.017	52.22 ± 33.83	65.33 ± 31.37	0.346
Grip strength (kg)	28.33 ± 6.64	22.42 ± 6.69	<0.001	16.55 ± 4.05	15.66 ± 4.57	0.355
ADL (score)	0.29 ± 1.21	1.79 ± 2.66	<0.001	0.18 ± 0.71	1.18 ± 2.14	0.004
IADL (score)	1.08 ± 2.27	3.63 ± 4.22	<0.001	0.70 ± 1.56	3.27 ± 3.65	<0.001

Mean ± SD. PA, physical activity; ADL, activities of daily living; IADL, instrumental activities of daily living.

A one-way ANOVA revealed that, among male individuals, the normal cognitive group exhibited significantly higher values for PA time (*p* < 0.05), grip strength (*p* < 0.001), ADLs (*p* < 0.001), and IADL (*p* < 0.001) compared to the cognitive impairment group. In contrast, among female individuals, ADLs (*p* < 0.01) and IADLs (*p* < 0.001) were significantly higher in the normal cognitive group compared to the cognitive impairment group. No significant differences were observed in PA frequency across both gender groups, and similarly, PA time and grip strength did not exhibit significant differences in the female groups.

### 3.2 Associations between physical activity and cognitive function

Tables 4, 5 show the independent associations between physical activity and cognitive function among older adults participants with disabilities.

**Table 4:** Independent associations of objectively measured physical activity time with cognitive impairment in older adults adults with disabilities.

	Unadjusted		Adjusted^a^	
	OR (95% CI)	*p*-value	OR (95% CI)	*p*-value
Physical activity				
Engaging in 150 min per week	1		1	
Not engaging in 150 min per week	2.29 (1.32–3.97)	0.003	2.08 (1.13–3.83)	0.018

OR, odds ratio; CI, confidence interval; PA, physical activity.

a Adjusted for sex (male and female), body mass index (<25 and ≥ 25), smoke (smoker and non-smoker), and alcohol (drinkers and non-drinkers).

**Table 5:** Independent associations of participation in regular physical activity with cognitive impairment in older adults adults with disabilities.

	Unadjusted		Adjusted^a^	
	OR (95% CI)	*p*-value	OR (95% CI)	*p*-value
Regular PA				
Participation in regular PA	1		1	
Not participating in regular PA	1.22 (1.08–1.38)	0.002	1.116 (1.01–1.34)	0.033

OR, odds ratio; CI, confidence interval; PA, physical activity.

a Adjusted for sex (male and female), body mass index (<25 and ≥ 25), smoke (smoker and non-smoker), and alcohol (drinkers and non-drinkers).

In the unadjusted model, participants who met the recommended PA time (OR = 2.29, 95% CI = 1.32–3.97) demonstrated significantly higher levels of cognitive function compared to those who did not meet the recommended PA times. Those failing to meet the recommended PA duration were 2.29 times more likely to exhibit cognitive decline. After adjusting for covariates such as sex, body mass index, smoking status, and alcohol consumption, the association between meeting the recommended PA duration and cognitive function was attenuated (OR = 2.08, 95% CI = 1.13–3.83). Nevertheless, even after adjustment, older adults Korean adults with disabilities who did not meet the recommended PA durations remained 2.08 times more likely to experience cognitive decline (Table 4).

Similarly, in the unadjusted model, participants who engaged in PA at least one day per week (OR = 1.22, 95% CI = 1.08–1.38) were more likely to exhibit higher levels of cognitive function compared to those who did not engage in PA at least one day per week. Individuals not engaging in PA at least one day per week were 1.22 times more likely to show signs of cognitive decline. Upon adjusting for covariates (sex, body mass index, smoking status, and alcohol consumption), the strength of this association diminished slightly (OR = 1.116, 95% CI = 1.01–1.34). Even with these adjustments, older adults Korean adults with disabilities who did not engage in PA at least one day per week remained 1.116 times more likely to experience cognitive decline (Table 5).

### 3.3 Correlations coefficients between K-MMSE scores and grip strength

**Figure 2:**
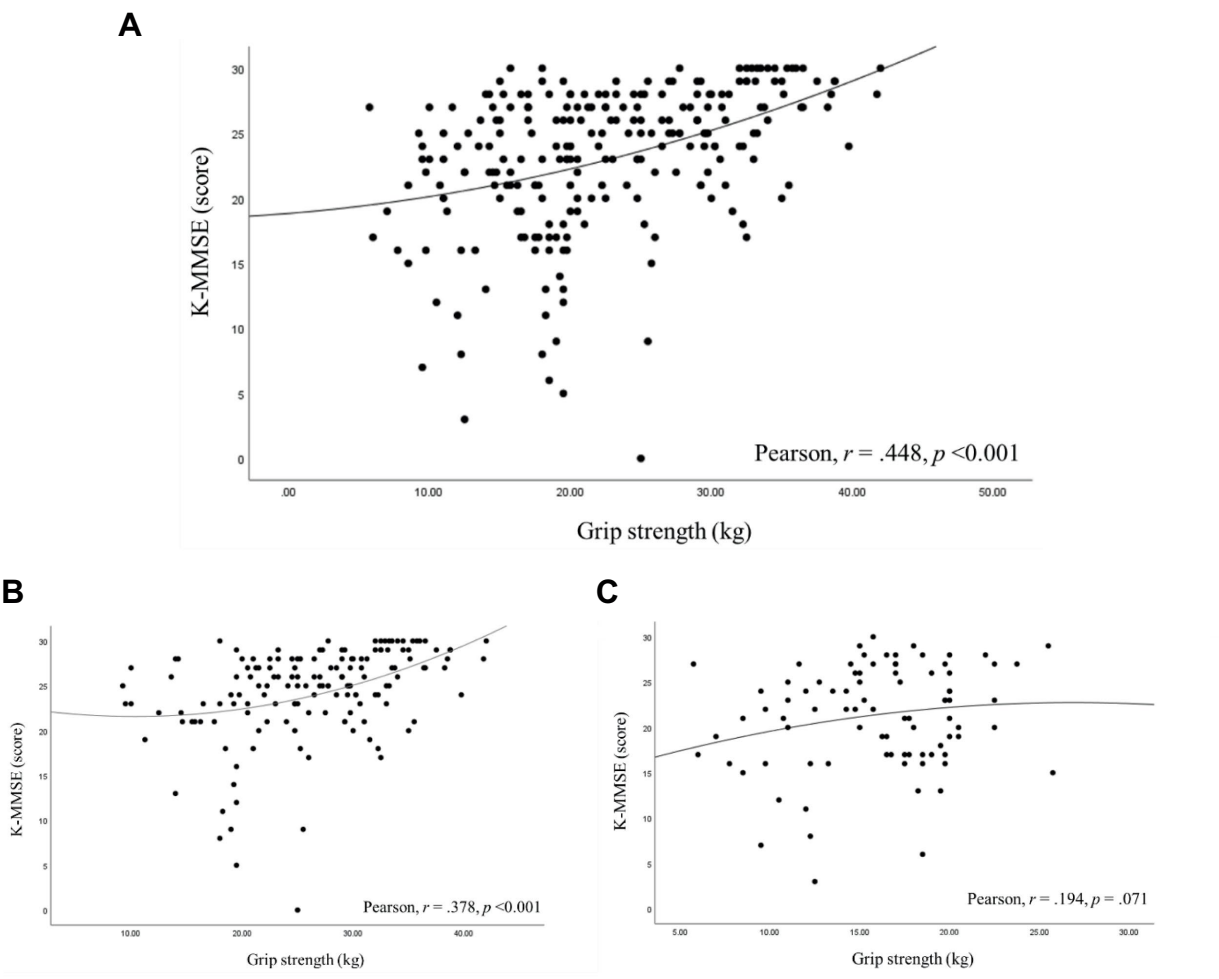
Pearson’s correlation coefficients between the cognitive score and grip strength. **(A)** all participants, **(B)** male participants, **(C)** female participants.

Figure 2 shows the correlation coefficients between K-MMSE scores and grip strength. A significant positive correlation was observed between K-MMSE scores and grip strength across all participants (r = 0.448, *p* < 0.001). In the male participant group, a significant positive correlation was also noted between K-MMSE scores and grip strength (r = 0.378, p < 0.001). Conversely, no significant correlation was found in the female participant group.

## 4 Discussion

In this study, we found that older adults Korean adults with disabilities who did not meet the recommended PA time exhibited a decline in cognitive function that was approximately 2.29 times greater compared to those who met the recommended PA time. Similarly, participants who did not engage in PA at least once a week experienced a decline in cognitive function that was approximately 1.22 times greater compared to those who did engage in PA at least once a week. A positive correlation was also found, wherein K-MMSE scores increased with greater grip strength. Furthermore, ADL and IADL scores were lower in the group with normal cognitive function compared to the group with cognitive impairment.

Approximately 1.5 billion people globally live with some form of physical, mental, sensory, or intellectual disability. These individuals are 16–62% less likely to meet established physical activity guidelines and are consequently at a higher risk of experiencing severe health issues associated with inactivity compared to those without disabilities (17). Research has shown that individuals with disabilities who do not meet recommended levels of physical activity report a significantly lower health-related quality of life than their non-disabled counterparts who are physically active. Importantly, these associations persist even after adjusting for potential confounders (18). Disabilities are often influenced by mental and neuromusculoskeletal dysfunction, factors that also contribute to lower energy expenditure during periods of physical activity. These relationships hold true even after accounting for age and environmental factors (19). Physical activity has been shown to lower the risk of disability due to all causes in both men and women. Those who engage in physical activity at sufficient volumes experience a notably reduced risk of disability (20). Physical activity is particularly crucial for older adults individuals who have chronic conditions or disabilities (21). Our study provides compelling evidence that older adults individuals with disabilities who did not meet recommended physical activity durations were 2.29 times more likely to experience cognitive decline than those who did meet these guidelines. Additionally, those who did not engage in physical activity at least once a week were 1.22 times more likely to exhibit cognitive decline. These findings underscore the importance of physical activity in maintaining and even enhancing cognitive function and neural circuitry in the older adults, especially in tasks that rely on the prefrontal cortex and hippocampus (22). Previous research has linked MVPA to dorsolateral prefrontal cortex volume. Specifically, the duration of MVPA has been correlated with preserved gray matter volume in frontal brain regions (23). Furthermore, one study found that greater walking activity at baseline, as assessed through a validated questionnaire, predicted increased total hippocampal volume after 9 years (24). Animal studies have also demonstrated increases in prefrontal cortex and hippocampus volume, suggesting that the formation of new cells is facilitated by increased nutrient supply via new vasculature (25). Activities such as running have been found to encourage the proliferation and survival of new neurons in the hippocampus (26). Regular engagement in PA, as well as increased physical activity durations, can have a significantly positive impact on improving cognitive function among the older adults with disabilities.

While numerous methods are available for studying the decline in muscle strength associated with aging, grip strength is often favored as an index due to its ease of assessment, affordability, high feasibility, and the validity of the data it provides (27). Previous research has indicated a link between muscle strength and overall cognitive function. A study involving 492 women found that both low gait speed (OR: 2.42, 95% CI: 1.72–3.40) and low handgrip strength (OR: 1.81, 95% CI: 1.33–2.46) were associated with cognitive impairment (28). Another study with 207 older adults participants aged 85 years and older revealed a significant correlation between Mini-Mental State Examination (MMSE) scores and handgrip strength, as determined by the simple regression analysis (29). Furthermore, a cross-sectional study comprising 1,396 older adults participants with a mean age of 77 years established that increased grip strength was positively correlated with performance in IADL (r = 0.28; *p* < 0.001) as well as Montreal Cognitive Assessment scores (*r* = 0.28; *p* < 0.001) (30). Our study is consistent with these findings, revealing a relationship between handgrip strength and cognitive function in older adults individuals with disabilities (*r* = 0.448, *p* < 0.001). Moreover, among male older adults participants with disabilities, those in the cognitively normal group exhibited significantly higher grip strength compared to those in the cognitive impairment group. This contrast was not statistically significant among female older adults participants with disabilities; however, grip strength tended to be higher in the cognitively normal group. The lack of a significant correlation between handgrip strength and cognitive function in female older adults with disabilities might be attributable to differing patterns of disability compared to their male counterparts.

Furthermore, muscle mass, an indicator of muscle strength, is correlated with dependency in ADLs and IADLs (31). In addition to muscle strength, the severity of cognitive impairment also significantly affects an individual’s ability to perform ADLs and IADLs. Specifically, individuals with mild or moderate cognitive impairments are at a higher risk of functional loss compared to those with unimpaired cognitive abilities (32). ADLs and IADLs serve as metrics for gaging an individual’s capacity for social interaction and independent living, thus functioning as critical indicators for assessing the necessary level of daily activities for familial and social life (33). In this context, a higher score in ADLs and IADLs implies an increased dependency, indicating that older adults individuals are unable to live independently and require assistance. In the present study, we observed significant differences in ADL and IADL scores between the two groups, with lower scores noted in the normal cognitive group compared to the cognitively impaired group. Previous research has shown a link between dependency in ADLs and IADLs and various factors such as length of hospital stay, frequency of hospital visits, and the presence of co-morbidities such as dementia (33). Physical activity and exercise serve as both preventive and therapeutic factors, mitigating the risk of physical and mental disabilities, thereby promoting independence in daily life (34). Regular physical activity has been shown to stave off chronic diseases by enhancing muscle activity and reducing inflammatory biomarkers in the older adults population (35). Therefore, physical activity could potentially improve ADL and IADL performance, thereby decreasing the likelihood of ADL and IADL incompetence among the older adults population (36). This protective effect of physical activity on ADL disability appears to be mediated through complex and likely multifactorial pathways (37).

The present study had some limitations that warrant attention. First, the assessment of physical activity and participant involvement relied on self-reported questionnaire data, which may introduce bias. Second, although disability classifications were determined by medical doctors, the study did not investigate the relationship between these classifications and cognitive function. Each type of disability may have its own set of movement limitations, and for this reason, the association with cognitive function may be different for different types of disabilities. Third, the study’s cross-sectional design based on a national survey limits its ability to establish causal relationships. Intervention studies are needed to determine whether decreased physical activity in older adults individuals with disabilities affects cognitive function or whether decreased cognitive function leads to decreased physical activity. Finally, frailty has been reported to have a significant impact on cognitive decline in the older adults (38). The older adults with disabilities are much more likely to be exposed to frailty than the older adults without disabilities because they have more limiting factors. Future studies should aim for more objective measures of physical activity, nuanced categorization of disorders, and the incorporation of long-term longitudinal designs.

## 5 Conclusion

This study highlighted the relationship between cognitive function and levels of physical activity among older adults individuals with disabilities. Our findings offer robust evidence that older adults individuals with disabilities who do not meet recommended guidelines for PA time, and who engage in PA only sporadically, face higher odds ratios for cognitive decline compared to those who are more active. A previous study indicated that older adults individuals without disabilities who fail to meet recommended PA levels are 1.63 times more likely to experience cognitive decline (10). In the population of older adults individuals with disabilities, the odds ratio is even more pronounced (OR = 2.29, 95% CI = 1.32–3.97), underscoring the importance of increased PA for this group. Additionally, we found that muscle strength is positively correlated with cognitive function, which in turn impacts activities of daily living. Therefore, we strongly advocate for enhancing both the amount and frequency of physical activity as a strategy to mitigate cognitive decline and improve daily living activities for older adults individuals with disabilities.

## Data availability statement

The datasets presented in this study can be found in online repositories. The names of the repository/repositories and accession number(s) can be found here: https://survey.keis.or.kr/klosa/klosa04.jsp.

## Ethics statement

Written informed consent was not obtained from the individual(s) for the publication of any potentially identifiable images or data included in this article because the Korean Longitudinal Study of Aging was approved by the research ethics committee of the KLoSA. The survey data are publicly available and can be downloaded from the employment survey site with personal information removed.

## Author contributions

S-TL: Conceptualization, Data curation, Formal analysis, Methodology, Writing – original draft. H-BK: Data curation, Formal analysis, Writing – original draft. J-HK: Conceptualization, Data curation, Writing – original draft. EC: Conceptualization, Data curation, Writing – original draft. K-LJ: Conceptualization, Data curation, Writing – original draft. H-JP: Conceptualization, Data curation, Writing – original draft. D-HP: Conceptualization, Data curation, Formal analysis, Methodology, Writing – original draft.
